# Health care costs of incident ADHD in children and adolescents in Germany – A claims data analysis within the framework of the consortium project INTEGRATE-ADHD

**DOI:** 10.25646/12303

**Published:** 2024-09-18

**Authors:** Lena Hasemann, Katharina Weinert, Jana Diekmannshemke, Robert Schlack, Ann-Kristin Beyer, Anne Kaman, Ulrike Ravens-Sieberer, Marcel Romanos, Thomas Jans, Peter Heuschmann, Cordula Riederer, the INTEGRATE-ADHD Study Group, Julian Witte

**Affiliations:** 1 Vandage GmbH, Bielefeld, Germany; 2 Robert Koch Institute, Department of Epidemiology and Health Monitoring, Berlin, Germany; 3 University Medical Centre Hamburg-Eppendorf, Department of Child and Adolescent Psychiatry, Psychotherapy and Psychosomatics, Research Section ‘Child Public Health’, Hamburg, Germany; 4 University Hospital Würzburg, Centre of Mental Health, Department of Child and Adolescent Psychiatry, Psychosomatics, and Psychotherapy, Würzburg, Germany; 5 University of Würzburg, Institute of Clinical Epidemiology and Biometry, Würzburg, Germany; 6 University Hospital Würzburg, Clinical Trial Centre, Würzburg, Germany; 7 University Hospital Würzburg, Institute for Medical Data Sciences, Würzburg, Germany; 8 DAK-Gesundheit, Hamburg, Germany

**Keywords:** Adolescent, Child, Attention-deficit disorder with hyperactivity, Financial stress, Propensity score, Health care costs, Delivery of health care, Comorbidity, Multivariate analysis, Insurance, Health

## Abstract

**Background:**

Attention-deficit/hyperactivity disorder (ADHD) is associated with increased costs for the family, the health care system and the society. Previous cost-of-illness studies in Germany usually focused on prevalent ADHD. This study addressed the research gap on health care resource utilisation and costs of children and adolescents with incident ADHD diagnosis using nationwide claims data from the statutory health insurance DAK-Gesundheit.

**Methods:**

A matched-control design (propensity score matching, 1:3 ratio) was used to examine the health care costs of incident ADHD patients compared with a non-ADHD control group, considering an observation period of four quarters. Besides bivariate statistics, multivariate analyses of total costs were used to consider relevant covariates.

**Results:**

Total health care costs for children and adolescents with ADHD in the first year after diagnosis exceeded those of the control group by € 1,505.3. According to the multivariate analysis, the group with incident ADHD had significantly higher (2.86-fold) health care costs when compared with non-ADHD peers. Sensitivity analyses proved these findings. In addition, the analyses identified children’s age and comorbidity index to be significantly associated with increased costs.

**Conclusions:**

ADHD in children and adolescents is associated with a significant economic burden. The results emphasise the need for social awareness, prevention, appropriate treatment and research efforts.

## 1. Introduction

Attention-deficit/hyperactivity disorder (ADHD) is a common neurodevelopmental disorder in children and adolescents with a prevalence rate of 5 % worldwide [1] and about 4 % in Germany [2-4]. ADHD is characterised by the core symptoms inattention, hyperactivity and/or impulsivity. Boys are more frequently affected than girls [5, 6]. The disease is associated with impairments in social and school life as well as a reduced overall quality of life [7]. Moreover, children and adolescents diagnosed with ADHD show higher risks for (psychiatric) comorbidities [8, 9] and may exhibit persistent symptoms and impairments in adulthood [10]. The broad impact of ADHD causes increased costs for the families, the health care system and the society. Compared with non-ADHD peers, ADHD patients show both higher direct health care costs and indirect costs, e.g. due to (parental) productivity losses [11, 12]. In 2019, the annual economic burden of ADHD in high-income countries is estimated to range from $ 831 to $ 20,538 per person [13]. Individual impairment, lifelong consequences and societal costs underline the public health relevance of ADHD. Systematic analyses of the costs related to ADHD are essential for the investigation of health care resource utilisation (HCRU) and provide valuable insights for health policy decision-making [14, 15].

**ADHD in Germany – Comparison and integration of administrative and epidemiological ADHD diagnostic data through clinical assessment (INTEGRATE-ADHD)**

**Consortium partners:** Robert Koch Institute Berlin, Department of Epidemiology and Health Monitoring, Germany; University Hospital Würzburg, Department of Child and Adolescent Psychiatry, Psychosomatics and Psychotherapy, Germany; University Medical Centre Hamburg-Eppendorf, Department of Child and Adolescent Psychiatry, Psychotherapy and Psychosomatics, Research Section ‘Child Public Health’, Germany; Vandage GmbH, Germany; University of Würzburg, Germany, Institute for Clinical Epidemiology and Biometry, Germany; DAK-Gesundheit, Germany

**Data holder:** Robert Koch Institute

**Objectives:** Identification of potential causes for the discrepancies between administrative ADHD diagnostic data (based on health insurance claims data) and epidemiological ADHD diagnostic data (based on surveys) for Germany, integration and validation of these data through a guideline-based clinical examination

**Study design:** Cross-sectional online survey, additional clinical examination of a sub-sample, data linkage with administrative health insurance data

**Population:** Children and adolescents who were insured with DAK-Gesundheit in 2020 and who were 0 to 17 years old at that time and for whom an administrative ADHD diagnosis labelled as confirmed was available in at least one quarter

**Gross sample**: 24,880 children and adolescents insured with DAK-Gesundheit with an administrative ADHD diagnosis

**Net sample:** 5,461 surveyed parents, 202 clinically examined children and adolescents

**Data collection period:** October 2021 to August 2022 (online survey), January 2022 to January 2023 (online clinical examination)

More information in German at www.rki.de/integrate-adhd

Studies on the economic burden of ADHD in Germany are usually based on claims data from Statutory Health Insurances (SHIs). Most of the published analyses have focused on patients with prevalent ADHD [14-16]. Cost analyses in cohorts with prevalent ADHD can illustrate the impact on health care systems. To accurately determine the HCRU and costs associated with ADHD, it is necessary to compare similar patient groups with and without an ADHD diagnosis in order to attribute the observed differences to the ADHD diagnosis [17]. In one of the few available studies on the cost of illness in ADHD incidence, Klora et al. [18] analysed the costs and treatment patterns of incident ADHD patients diagnosed in 2007. The health care costs of the ADHD group exceeded the costs of the matched control group by € 1,236 for ages 0 to 5 years and by € 1,673 for ages 6 to 17 years, respectively.

Considering the large number of unfavourable outcomes, an early and effective treatment of ADHD is essential. Multimodal treatment, combining psychosocial interventions and pharmacotherapy, has become the international gold standard in ADHD management in children and adolescents [19-21]. Accordingly, health care practices in Germany have changed which is reflected in an increasing proportion of ADHD patients with multimodal treatment over the last decade [22]. Yet, evidence on direct costs of incident ADHD in recent years is scarce. There has also been no investigation of the additional HCRU and costs attributed to ADHD when factors such as socioeconomic characteristics, comorbidity, or regional care structures are considered. However, such data are essential for the further development of treatment guidelines and care planning. This study investigated health care costs for children and adolescents newly diagnosed with ADHD from administrative records. The hypothesis was that individuals with incident ADHD would have higher costs compared with a control group without ADHD. The study was conducted as part of the INTEGRATE-ADHD consortium project. The primary aim of this project was to compare administrative, epidemiological and clinical diagnostic data on ADHD by linking the data at the individual level. Details of the aims and the conduct of this project can be found in Schlack et al. [23] and Beyer et al. [24].

## 2. Methods

### 2.1 Data base and study population

The study was based on anonymised claims data from 575,801 children and adolescents aged 0 to 17 living in Germany and insured with DAK-Gesundheit between 2018 and 2020. Besides demographic characteristics, the claims data contain information on the individual’s HCRU (including treatments, prescriptions), costs and documented diagnoses [25]. Additional information and data sources were used to expand the dataset with potential confounding factors. These include the German Index of Deprivation (GISD) based on the INKAR database [26], classification of place of residence (rural/urban, based on the number of inhabitants according to the INKAR database [25]), the paediatric comorbidity index developed by Sun et al. [27] and parameters of access to health care in terms of regional ratios of physicians and therapists [28]. The GISD was developed to explain regional differences in health and has three categories (low, middle, high), with high scores indicating poorer regional socioeconomic situations [29]. The paediatric comorbidity index integrates 24 conditions into a single numerical index and provides a summary measure of disease burden [27]. Due to the restriction of the claims data to three-digit ICD-10 codes, some discrepancies in the calculation of the index could not be avoided. The regional ratios of physicians and therapists (general practitioners, paediatricians, medical and psychological psychotherapists, psychiatrists/psychologists for children and adolescents) per 100,000 inhabitants were extracted from the register of the Federal Association of SHI Physicians [28].

### 2.2 Study design

A matched control design was used to examine the direct health care costs of patients with incident ADHD diagnosed within the period between the first quarter of 2019 to the first quarter of 2020. Cases of incident ADHD were identified by a confirmed outpatient or primary inpatient diagnosis (ICD-10-GM-2022 F90.0-9) in at least one quarter of the year (M1Q criterion) and an individual disease-free pre-observation period of at least four quarters. These patients were compared with non-ADHD over an observation period of four quarters (including the index quarter of the diagnosis). The control group (ratio 1:3) was selected from the subgroup of individuals without a diagnosis of ADHD in 2018 to 2020 using propensity score matching (PSM) with replacement. Variables included in the propensity score were the children’s age, sex, individual paediatric comorbidity index, the regional socioeconomic situation (GISD), the urbanicity (urban/rural) and the regional ratio of doctors and therapists. The approach aims to ensure the comparability of the study groups in terms of these factors that may influence HCRU and associated costs. The PSM was performed using nearest neighbour matching with a caliper (0.1), which ensures that the distance between two matched individuals is not greater than the caliper. To diagnose the quality of the resulting sample, the covariate balance in the matched groups was assessed. As the study adopted a SHI-/payer perspective, direct cost components were related to inpatient and outpatient care, drugs, medical aids, remedies and rehabilitation. Indirect costs (e.g. parental productivity losses) and out-of-pocket payments were not included.

### 2.3 Sensitivity analysis

To address uncertainties and to improve the methodological quality of the study, sensitivity analyses were conducted. In sensitivity analysis A, extreme outliers (*n* = 2) in health care expenditures on pharmaceuticals, identified in the control group, were excluded before matching. Sensitivity analysis B applied a more restrictive case definition of incident ADHD. Children and adolescents were included in the ADHD group if they met the following criteria: two confirmed outpatient diagnoses in two different quarters (M2Q) or one inpatient primary diagnosis (M1Q) or one confirmed outpatient diagnosis (M1Q) with ADHD medication (German Anatomical Therapeutic Chemical (ATC)-Classification: N06BA04, N06BA09, N06BA02, N06BA12, N06BA21) and a disease-free, pre-observational period of at least four quarters.

### 2.4 Statistical analysis

In a first step, bivariate analyses (e.g. t-tests) were applied comparing the ADHD group and the control group in terms of baseline characteristics (age, sex, comorbidity index, GISD, regional structure, regional ratios of physicians/therapists) and health care costs. To account for the positively skewed distribution of health care costs, a generalized linear model (GLM, gamma distribution) was implemented to examine the effect of incident ADHD on total health care costs considering covariates which are potentially associated with higher costs. These covariates are concordant with the variables in the PSM. Thus, the regression model accounts for remaining differences between the ADHD and the non-ADHD group and allows for more robust estimate effects. The estimation equation then becomes:


ln(y_it_) = β_0_ + β_1_ADHD_it_+γ’X_it_+u_it_,

where *y_it_* denotes the health care costs for individual *i* in period *t*. The binary dummy variable *ADHD_it_* indicates whether an incident ADHD diagnosis has been documented for individual *i* in period *t*. The *1 x N* vector **X**_*it*_compromises all covariates used for calculating the propensity scores. An overview of the dependent and independent variables can be found in the annex (Annex Table 1). All analyses were performed using the open-source R Software [30] at a significance level of 5 %.

## 3. Results

The PSM included 10,036 incident ADHD patients and 536,798 potential controls and led to a final study population of 10,033 cases and 30,093 controls. Three ADHD patients could not be assigned to a matching partner. The mean age in the ADHD and control group was 10.2 (*SD:* ADHD group = 3.3, control group = 3.4) (Table 1). As expected, the proportion of boys (ADHD group = 71.7 %, control group = 72.6 %) was higher than girls. The comorbidity index was 2.2 (*SD* = 2.6) in both study groups. About two thirds of the children and adolescents lived in urban areas. Medium GISD was most frequent in both the ADHD (66.7 %) and the non-ADHD group (67.3 %). The baseline characteristics did not show any significant differences and thus confirmed the comparability of both groups after PSM.

Table 2 shows the health care costs of the study groups over the 12-month observation period. The increment corresponds to the mean difference in costs between the group with and without ADHD. SHI-expenditure on outpatient care, inpatient care and remedies were significantly higher in ADHD patients. The average cost of medication showed high standard deviations and were higher in the control group. In addition, expenditure on medical aids was significantly higher in the control group than in the ADHD group. The mean total SHI expenditure per capita in the group of children and adolescents with incident ADHD (€ 2,633.6) was significantly higher than in those without incident ADHD (€ 1,128.3). Thus, the incremental costs of incident ADHD were € 1,505.3. The largest proportions of incremental costs were incurred by inpatient (+ € 868.4, 57.7 %) and outpatient services (+ € 706.7, 47.0 %).

A GLM (Gamma distribution) was implemented to estimate the effect of incident ADHD on total annual health care costs, controlling for the variables included in the PSM (Table 3). To facilitate the interpretation of the coefficients, Table 3 to Table 5 include exponentiated coefficient estimates. Given that all variables other than the predictor variable of interest are fixed, the exponentiated coefficient reflects the ratio between the estimated health care costs for a one scale unit increase in the predictor variable and the costs associated with the baseline level of the predictor variable. Table 3 shows that children and adolescents with incident ADHD had significantly higher health care costs – almost threefold as high – than their peers without a diagnosis of ADHD. In addition, a one unit increase in the comorbidity index was associated with an increase in estimated health care costs of roughly 20 %. Also, the results show a significant difference in total costs for children with medium compared with low GISD. Children and adolescents from areas of moderate deprivation had 23 % lower costs than those from areas of low deprivation. Furthermore, significant but small effects are observable for age and the regional ratio of medical psychotherapists.

Sensitivity analysis A compared children and adolescents of the ADHD group (*n* =10,033) with an adapted non-ADHD group of 30,093 individuals. Descriptive analyses (Annex Table 2) show that, in contrast to the main analysis, the mean cost of medication was higher in ADHD patients than in controls (+ € 23.8). The incremental total health care costs of incident ADHD were € 1,574.4. According to the multivariate analysis (GLM, Gamma distribution, Table 4), incident ADHD led to 3.11-fold total health care costs when compared with the adapted control group. This confirmed the result of the main analysis model.

Sensitivity analysis B includes 5,674 ADHD patients and a matched control group of 17,016 children and adolescents. Comparisons of mean health care costs between the groups (Annex Table 3) did not reveal any significant differences from the main analysis. Individuals diagnosed with ADHD had significantly higher total health care costs (€ 3,244.1) than their non-ADHD peers (€ 1,168.7). In line with the main analysis, the GLM (Table 5) showed significantly higher health care costs for children and adolescents with an incident diagnosis of ADHD compared with the control group.

## 4. Discussion

The present analyses investigated the direct health care costs of children and adolescents with incident ADHD adopting a SHI/payer perspective. Based on a matched control design, the analyses provide robust results and confirm the initial hypothesis that incident ADHD patients have significantly higher costs than non-ADHD individuals. The incremental costs of ADHD in the first year after diagnosis were € 1,505.3. Multivariate analyses (GLM) were applied to account for age, sex, regional structure, GISD comorbidity and access to health care services. The presence of an incident ADHD diagnosis was significantly associated with increased total health care costs. Compared with non-ADHD peers, the ADHD group had 2.86-fold total health care costs. Sensitivity analyses, which considered extreme outliers in costs on pharmaceuticals (sensitivity analysis A) as well as an adapted case definition of incident ADHD (sensitivity analysis B), led to comparable results. Repeating the multivariate analyses based on these study groups showed that total health care costs were significantly higher in the ADHD group than in the control group. As a part of the INTEGRATE-ADHD project [23] these results define a baseline value for the economic burden of incident ADHD and provide a basis for further health economic investigations in this area. The results further extend the state of research in terms of multivariate analyses on the effect of ADHD on total health care costs considering a comprehensive set of relevant covariates, including a proxy for the individual’s socioeconomic characteristics, an index of paediatric comorbidity as well as access to health care services.

The results of this study are consistent with previous studies reporting significantly higher health care costs for ADHD patients compared with non-ADHD controls. Schein et al. [12] examined the direct health care costs attributable to prevalent ADHD in 2018, based on claims data from the US. The excess costs were $ 1,759 per child (5 –11 years) and $ 2,424 per adolescent (12 –17 years) with ADHD. However, the comparability of the results is limited due to differences in health care systems and data sources. While the present study is based on claims data from children and adolescents with SHI coverage, the US study [12] analysed claims data from insured individuals and also included literature-based estimates for uninsured individuals. Studies based on German SHI claims data found similar levels of expenditure. Klora et al. [16] used SHI claims data from 2006 to 2008 to examine cost differences between prevalent ADHD patients and a non-ADHD control group. The reported incremental annual costs for children and adolescents were € 1,430 (0 – 5 years), € 1,623 (6 –12 years) and € 1,286 (13 –17 years). The results on direct medical costs of prevalent ADHD reported by Libutzki et al. [15] were based on SHI claims data from 2009 to 2014. The excess costs per year for individuals with ADHD compared with those without ADHD were € 1,494 for children (0 – 12 years) and € 1,447 for adolescents (13 – 17 years). So far, only Klora et al. [18] have investigated the costs of incident ADHD patients in Germany. Analyses based on SHI claims data from 2006 to 2008 showed that the incremental costs of incident ADHD compared with a matched control group were € 1,236 for children (0 – 5 years) and € 1,673 for adolescents (6 – 17 years). The level of incremental costs of ADHD reported in previous studies is comparable to the current finding (€ 1,505.3).

In the studies described above [15, 16, 18] outpatient and inpatient services also accounted for the largest proportion of incremental costs. In contrast to the existing evidence [16, 18], the present study did not find significantly higher pharmaceutical expenditure in ADHD compared with non-ADHD children and adolescents. This can be explained by the high standard deviations in both groups, which are a multiple of the observed mean differences. These high standard deviations are in turn due to a few high-cost cases among the individuals. The application of GLMs accounted for the remaining differences between the matched groups in terms of individual characteristics and structural conditions. Apart from ADHD, age and more severe comorbidity were the most relevant covariates associated with higher health care costs. Results were mixed for GISD and regional ratios of physicians and psychotherapists. While many cost-of-illness studies have used descriptive and bivariate statistics [12, 15, 16, 18], Gupte-Singh et al. [11] used a two-part expenditure model to examine the economic burden of paediatric ADHD. Using data from the US Medical Expenditure Survey, they adjusted for individual factors and access to health care. The prevalent paediatric ADHD group with positive expenditures had 58.4 % higher expenditures compared with individuals without ADHD. Consistent with the current analysis, age and comorbidity burden were significantly associated with higher health care costs (second part of the regression model). The researchers also found significant coefficients for ethnicity and health care insurance status.

This study has several strengths and limitations. A major strength is the large sample of 575,801 children and adolescents (0 –17 years) insured with DAK-Gesundheit, which formed the basis of this study and which can be considered representative of the German SHI population in terms of age and gender [31]. The SHI claims data contain a relevant set of individual characteristics and factors potentially associated with health care costs, which were included in the PSM and the multivariate analysis. Furthermore, two sensitivity analyses were performed to address uncertainties (e.g. in case definition) and to improve the methodological quality. Thus, the study provides robust results on the health care costs of incident ADHD in children and adolescents in Germany. However, some limitations should be considered when interpreting the results of claims data analyses. SHI claims data are not primarily collected for research purposes. Due to the administrative nature of the data, the identification of ADHD cases was based on documented diagnoses, which is likely to lead to a systematic underestimation because a large number of patients remain unrecorded [15]. As the dataset was restricted to three-digit ICD-10 codes, deviations from the specification of the comorbidity index [27] were unavoidable. Furthermore, the GISD index is a proxy for individual socioeconomic status based on regional data. It does not provide information at the individual level. The relatively short pre-observation period may have led to an overestimation of ADHD incidence in the sample [32]. Future studies should address these limitations and extend the periods of (pre-)observation. Other potential improvements relate to individual-level information on socioeconomic status and access to health care. Given the broad impact of ADHD, a societal perspective of cost analyses may be valuable. The current study provides a baseline value for the health care costs of ADHD in children and adolescents and does not focus on details in patient pathways (e.g. utilisation of pharmacotherapy or multimodal treatment). Thus, future research should examine the determinants of health care costs and aspects of time in HCRU in ADHD patients to inform the development of intervention strategies. In addition, the INTEGRATE-ADHD project analyses potential differences in administrative, epidemiological and clinical diagnostic data [23]. One of the main health economic analyses based on the INTEGRATE-ADHD dataset will consider the subsample of children and adolescents who participated in a guideline-based clinical diagnostics. It will examine whether children and adolescents with an administrative and clinically validated diagnosis of ADHD have higher health care costs than those with an administrative but clinically non-validated diagnosis.

Overall, the analyses provide evidence of the economic burden of ADHD in children and adolescents in the first year after the initial diagnosis. The results emphasise the need for social awareness, prevention, appropriate treatment and research efforts, not only from a patient and carer perspective but also from the perspective of the health system.

## Key statements

- The incremental direct costs of incident ADHD were € 1,505.3.
- In multivariate analysis, the costs of children and adolescents with incident ADHD were almost threefold higher than those of their non-ADHD peers.
- The economic burden of ADHD highlights the need for social awareness, prevention, appropriate treatment and research efforts.

## Figures and Tables

**Table 1:** Sample characteristics (*N* = 40,126; *n* =11,088 female, *n* = 29,038 male). Source: DAK-Gesundheit

	ADHD Group (*n* =10,033)	Control Group (*n* = 30,093)	*p*-value
Age mean *(SD)*	10.2 (3.3)	10.2 (3.4)	0.922
**Sex (%)**			
Female	2,840 (28.3)	8,248 (27.4)	0.084
Male	7,193 (71.7)	21,845 (72.6)	
Comorbidity Index mean *(SD)*	2.2 (2.6)	2.2 (2.6)	0.051
**GISD (%)**			
Low	1,249 (12.5)	3,626 (12.1)	0.481
Middle	6,696 (66.7)	20,256 (67.3)	
High	2,088 (20.8)	6,211 (20.6)	
**Regional structure (%)**			
Urban	6,596 (65.7)	19,916 (66.2)	0.429
Rural	3,437 (34.3)	10,177 (33.8)	
Regional ratio of general practitioners mean *(SD)*	67.0 (5.0)	67.0 (5.0)	0.952
Regional ratio of paediatricians mean *(SD)*	9.8 (1.4)	9.8 (1.4)	0.786
Regional ratio of medical psychotherapists mean *(SD)*	7.6 (3.8)	7.6 (3.8)	0.583
Regional ratio of psychological psychotherapists mean *(SD)*	38.7 (15.9)	38.9 (16.0)	0.352
Regional ratio of psychiatrists or psychologists for children and adolescents mean *(SD)*	1.4 (0.6)	1.4 (0.6)	0.9 41

*SD* = standard deviation, ADHD = attention-deficit/hyperactivity disorder, GISD = German Index of Deprivation

**Table 2:** Health care costs of the ADHD group compared with the control group (*N* = 40,126; *n* =11,088 female, *n* = 29,038 male). Source: DAK-Gesundheit

	ADHD Group (*n* =10,033)	Control Group (*n* = 30,093)	*p*-value	Increment (ADHD Group vs. Control Group)
Outpatient care mean € *(SD)*	1,044.2 (1,183.8)	337.5 (639.7)	< 0.001^*^	706.7
Inpatient care mean € *(SD)*	1,205.3 (6,082.7)	336.9 (2,833.3)	< 0.001^*^	868.4
Pharmaceuticals mean € *(SD)*	252.1 (2,888.0)	286.4 (7,200.6)	0.498	-34.3
Medical aids mean € *(SD)*	56.4 (488.1)	138.3 (1,287.5)	< 0.001^*^	-82.0
Remedies mean € *(SD)*	63.6 (105.1)	20.4 (67.0)	< 0.001^*^	43.2
Rehabilitation mean € *(SD)*	12.0 (695.7)	8.8 (426.4)	0.660	3.3
**Total health care costs mean € *(SD)***	**2,633.6** **(7,071.2)**	**1,128.3** **(8,205.3)**	< 0.001^*^	**1,505.3**

^*^Significance level *p* < 0.05

*SD* = standard deviation, ADHD = attention-deficit/hyperactivity disorder

**Table 3.** Results of the regression analysis (GLM, Gamma distribution) on total health care costs (*N* = 40,126; *n* =11,088 female, *n* = 29,038 male). Source: DAK-Gesundheit

	Exp (Coefficient)	(95 % CI)	*p*-value
Intercept	180	(43.3 – 757)	< 0.001^*^
ADHD	**2.86**	**(2.40 – 3.44)**	< 0.001^*^
Age	1.03	(1.00 – 1.05)	0.021^*^
Sex: male (reference female)	1.14	(0.95 – 1.35)	0.141
Comorbidity Index	1.22	(1.18 – 1.26)	< 0.001^*^
GISD: medium (reference GISD low)	0.77	(0.59 – 0.98)	0.037^*^
GISD: high (reference GISD low)	0.88	(0.65 – 1.19)	0.408
Regional structure: rural (reference urban)	1.08	(0.88 – 1.32)	0.500
Ratio of general practitioners	1.01	(0.99 – 1.03)	0.362
Ratio of paediatricians	1.05	(0.98 – 1.12)	0.180
Ratio of medical psychotherapists	0.95	(0.91 – 1.00)	0.050^*^
Ratio of psychological psychotherapists	1.01	(1.00 – 1.02)	0.091
Ratio of psychiatrists or psychologists for children and adolescents	0.90	(0.77 – 1.04)	0.157

GISD = German Index of Deprivation, Exp(Coefficient) = exponentiated coefficient estimate, CI = Confidence interval, ADHD = attention-deficit/hyperactivity disorder

^*^Significance level *p* < 0.05

**Table 4:** Sensitivity analysis A: results of the regression analysis (GLM, Gamma distribution) on total health care costs(*N* = 40,126; *n* =11,085 female, *n* = 29,041 male). Source: DAK-Gesundheit

	Exp (Coefficient)	(95 % CI)	*p*-value
Intercept	101	(42.2 – 244)	< 0.001^*^
ADHD	**3.11**	**(2.78 – 3.48)**	< 0.001^*^
Age	1.03	(1.01 – 1.04)	< 0.001^*^
Sex: male (reference female)	1.08	(0.96 – 1.20)	0.179
Comorbidity Index	1.23	(1.21 – 1.26)	< 0.001^*^
GISD: medium (reference GISD low)	1.03	(0.88 – 1.20)	0.712
GISD: high (reference GISD low)	1.07	(0.89 – 1.29)	0.477
Regional structure: rural (reference urban)	0.99	(0.87 – 1.13)	0.913
Ratio of general practitioners	1.01	(1.00 – 1.03)	0.052
Ratio of paediatricians	1.07	(1.03 – 1.12)	0.0 01^*^
Ratio of medical psychotherapists	0.95	(0.92 – 0.98)	< 0.001^*^
Ratio of psychological psychotherapists	1.01	(1.00 – 1.01)	0.072
Ratio of psychiatrists or psychologists for children and adolescents	0.90	(0.82 – 0.99)	0.033^*^

GISD = German Index of Deprivation, Exp(Coefficient) = exponentiated coefficient estimate, CI = Confidence interval, ADHD = attention-deficit/hyperactivity disorder

^*^Significance level *p* < 0.05

**Table 5:** Sensitivity analysis B: results of the regression analysis (GLM, Gamma distribution) on total health care costs(*N* = 22,690; *n* = 6,100 female, *n* =16,590 male). Source: DAK-Gesundheit

	Exp (Coefficient)	(95 % CI)	*p*-value
Intercept	458	(63.70 – 3308)	< 0.001^*^
ADHD	**3.44**	**(2.70 – 4.41)**	**< 0.001**
Age	1.04	(1.00 – 1.07)	0.030^*^
Sex: male (reference female)	1.16	(0.91 – 1.46)	0.229
Comorbidity Index	1.20	(1.16 – 1.26)	< 0.001^*^
GISD: medium (reference GISD low)	0.65	(0.46 – 0.91)	0.011 ^*^
GISD: high (reference GISD low)	0.74	(0.49 – 1.10)	0.136
Regional structure: rural (reference urban)	1.18	(0.90 – 1.56)	0.231
Ratio of general practitioners	1.00	(0.97 – 1.03)	0.983
Ratio of paediatricians	1.00	(0.91 – 1.10)	0.992
Ratio of medical psychotherapists	0.98	(0.92 – 1.04)	0.426
Ratio of psychological psychotherapists	1.01	(1.00 – 1.02)	0.150
Ratio of psychiatrists or psychologists for children and adolescents	0.96	(0.78 – 1.19)	0.698

GISD = German Index of Deprivation, Exp(Coefficient) = exponentiated coefficient estimate, CI = Confidence interval, ADHD = attention-deficit/hyperactivity disorder

^*^Significance level *p* < 0.05

**Annex Table 1:** Overview on (in-)dependent variables. Source: DAK-Gesundheit

Independent Variables	Explanation
**Age**	Years of age in index quarter
**Sex**	Male/female
**Comorbidity Index** ► validated measurement of pediatric comorbidity [27]	
**German Index of Socioeconomic Deprivation (GISD)** ► measurement of socioeconomic deprivation using information on the education, employment and income situation in districts and municipalities from the INKAR database [29]	Low/medium/high
**Regional structure**	Urban area/rural area
**Regional ratio of general practitioners**	Regional number of providers in relation to the regional population (per 100,000 inhabitants)
**Regional ratio of paediatricians**	
**Regional ratio of medical psychotherapists**	
**Regional ratio of psychological psychotherapists**	
**Regional ratio of psychiatrists or psychologists for children and adolescents**	
**ADHD (incident, main analysis)** ► confirmed outpatient or primary inpatient diagnosis (ICD-10-GM-2022 F90., incl. F90.0, F90.1, F90.8, F90.9) in at least one quarter of the year (M1Q) ► disease-free pre-observation period of at least four quarters	Yes/no
**ADHD (incident, sensitivity analysis B)** ► two confirmed outpatient diagnoses (M2Q) or inpatient primary diagnosis (M1Q) or confirmed outpatient diagnosis (M1Q) and ADHD medication ► disease-free pre-observation period of at least four quarters	Yes/no
**Dependent Variables**	**Explanation**
**Health care costs**	SHI-expenditure (€) on health care services within the period of observation (twelve months)
Outpatient care	
Inpatient care	
Pharmaceuticals	
Medical aids	
Remedies	
Rehabilitation	
**Total health care costs**	Total SHI-expenditure (€) on health care services within the period of observation (twelve months)

ADHD = attention-deficit/hyperactivity disorder, INKAR = Indikatoren und Karten zur Raum-und Stadtentwicklung, ICD-10 = International Statistical Classification of Diseases and Related Health Problems, 10th Revision, SHI = statutory health insurance

**Annex Table 2:** Sensitivity analysis A: Health care costs of the ADHD group compared to the control group (*N* = 40,126; *n* =11,085 female, *n* = 29,041 male). Source: DAK-Gesundheit

	ADHD Group (*n* =10,033)	Control Group (*n* = 30,093)	*p*-value	Increment (Cases vs. Controls)
**Outpatient care** mean € *(SD)*	1,044.2 (1,183.8)	335.4 (628.5)	< 0.001^*^	708.8
**Inpatient care** mean € *(SD)*	1,205.3 (6,082.7)	330.5 (2,800.0)	< 0.001^*^	874.8
**Pharmaceuticals** mean € *(SD)*	252.1 (2,888.0)	228.4 (3,994.8)	0.520	23.8
**Medical aids** mean € *(SD)*	56.4 (488.1)	135.9 (1,266.9)	< 0.001^*^	-79.5
**Remedies** mean € *(SD)*	63.6 (105.1)	20.4 (67.0)	< 0.001^*^	43.1
**Rehabilitation** mean € *(SD)*	12.0 (695.7)	8.6 (425.8)	0.644	3.4
**Total health care costs** mean € *(SD)*	**2,633.6** **(7,071.2)**	**1,059.2** **(5,553.3)**	< 0.001^*^	**1,574.4**

^*^significance level *p* < 0.05

*SD* = standard deviation, ADHD = attention-deficit/hyperactivity disorder

**Annex Table 3:** Sensitivity analysis B: Health care costs of the ADHD group compared to the control group (*N* = 22,690; *n* = 6,100 female, *n* =16,590 male). Source: DAK-Gesundheit

	ADHD Group (*n* = 5,674)	Control Group (*n* =17,016)	*p*-value	Increment (Cases vs. Controls)
**Outpatient care** mean € *(SD)*	1,217.1 (1,227.3)	336.6 (644.8)	< 0.001^*^	880.5
**Inpatient care** mean € *(SD)*	1,586.8 (7,11 6.3)	337.3 (2,660.1)	< 0.001^*^	1,249.5
**Pharmaceuticals** mean € *(SD)*	303.6 (3,222.1)	317.0 (8,687.1)	0.865	-13.5
**Medical aids** mean € *(SD)*	57.0 (516.8)	147.6 (1,371.2)	< 0.001^*^	-90.6
**Remedies** mean € *(SD)*	72.1 (11 0.6)	20.4 (68.2)	< 0.001^*^	51.8
**Rehabilitation** mean € *(SD)*	7.5 (209.5)	9.8 (420.2)	0.598	-2.2
**Total health care costs** mean € *(SD)*	**3,244.1** **(8,055.4)**	**1,168.7** **(9,479.2)**	< 0.001^*^	**2,075.4**

^*^significance level *p* < 0.05

*SD* = standard deviation, ADHD = attention-deficit/hyperactivity disorder
